# The effect of integration of medical and preventive services on the prevention and control of public health emergencies in China

**DOI:** 10.3389/fpubh.2026.1805192

**Published:** 2026-06-24

**Authors:** Minghua Zhou

**Affiliations:** Department of Administration Office, Luzhou People's Hospital, Luzhou, Sichuan, China

**Keywords:** integration of medical and preventive services, medical resources, prevention and control effect, public health emergencies, public health resources

## Abstract

**Objective:**

To analyze the effect of integration of medical and preventive services on the prevention and control of public health emergencies in China, and to provide a scientific basis for improving prevention and control capabilities.

**Methods:**

The coupling coordination degree was used to calculate the comprehensive scores for the integration of medical and preventive services in 31 provincial-level administrative areas from 2018 to 2023. The incidence rates of Category A and B infectious diseases were selected as the explained variables for prevention and control effectiveness, and a panel model was used to analyze the effect of these scores on the prevention and control of public health emergencies during the same period.

**Results:**

The comprehensive scores for the integration of medical and preventive services in China from 2018 to 2023 were 0.558, 0.567, 0.574, 0.578, 0.577, and 0.579, respectively. Areas such as Guangdong and Shandong had comprehensive scores above 0.94, while areas including Tibet, Qinghai, and Ningxia had comprehensive scores below 0.23. The comprehensive score for the integration of medical and preventive services in China was statistically significant at the 0.05 level (*t* = −1.102, *p* < 0.05), with a regression coefficient of −227.428. This indicates that the integration of medical and preventive services had a significant negative impact on the public health emergencies. The comprehensive score in the eastern region was statistically significant at the 0.01 level (*t* = −3.339, *p* < 0.01), with a regression coefficient of −703.139. This indicates that the eastern region had a significant negative impact on the public health emergencies. The central and western regions did not pass the significance level test.

**Conclusion:**

Regional disparities in the integration of medical and preventive services in China are relatively obvious. The integration of medical and preventive services significantly affected the prevention and control of public health emergencies. The effect of the integration of medical and preventive services on the prevention and control of public health emergencies showed regional differences.

## Introduction

Public health is a critical issue related to people's wellbeing, as well as social harmony and stability (1). The integration of medical and preventive services is a working mechanism that combines medical and public health resources to achieve coordinated disease prevention and treatment management. How this integration impacts public health, and specifically how it affects the effects of prevention and control of public health emergencies, which is a question worthy of in-depth research. In December 2023, the Guiding Opinion on Promoting High-Quality Development in Disease Prevention and Control [Guo Ban Fa (2023) No. 46] emphasized innovating mechanisms for medical and public health coordination and integration (2). The opinion called for advancing comprehensive collaboration between medical and public health institutions and establishing mechanisms for talent mobility, cross-training, service integration, and information sharing. The integration of medical and preventive services is an innovative model in the healthcare sector. Its core mission is to deeply integrate medical and public health services. This is accomplished by taking various measures, including establishing independent public health departments within medical institutions, building infectious disease surveillance networks centered on sentinel hospitals, creating one-stop service models such as integrated medical and public health clinics, forming integrated medical and public health service teams at the grassroots level to provide family doctor contract services, and establishing collaborative consultation mechanisms between medical institutions and disease control agencies. These efforts aim to shift the focus from a “disease-centered” approach to a “health-centered” one. In response to public health emergencies, disease prevention efforts must quickly identify the source of the infection, the transmission routes, and the high-risk populations. They must also develop targeted measures, and implement public health emergency responses. Medical services should improve tiered treatment protocols, enhance diagnostic and treatment capabilities, shorten hospital stays, and reduce pressure on medical resources (3). Therefore, when responding to public health emergencies, it is important to adhere to the principle of prioritizing prevention and integrating it with treatment, and to strengthen the integration of medical and preventive services. This integration plays an important role in enhancing the governance capacity for public health emergencies.

At present, studies on the integration of medical and preventive services and public health emergencies are primarily divided into three aspects. First, the integration of healthcare policies and public health emergency response analysis including effective policies of county and township health sector integration in China (4), physical-medical integration policies and health equity promotion in China (5), and public health surveillance of behavioral health emergencies through emergency medical service data (6). Second, the practice of integration of medical and public health services, including the systematic integration between medical, public health, and social entities at Cornell University (7), the dynamics of traditional, complementary and alternative medicine integration in the India public health system (8), and community health work and social work collaboration for integration in healthcare and public health settings in the United States (9). Third, the analysis of the mechanisms and influencing factors of the integration of medical and disease prevention, including the influencing factors and symbiotic mechanism of the integration of medical care and disease prevention during the COVID-19 pandemic in Zhejiang (10), the impact of Sanming's integrated medical service model on medical expenditures, service provision, and resource allocation in China (11), and the key factors influencing the vertical integration of electronic health records in Chinese medical consortiums (12). Currently, studies on the integration of medical and preventive services and public health emergencies primarily focus on the practical implementation of integrated medical and public health services, as well as the factors influencing such integration. Relevant initiatives have already been implemented in regions such as the United States and India. While China has conducted policy analyses and implemented practical models of integrated medical services at the county and township levels, it lacks empirical analysis on the effectiveness of the integration of medical and preventive services on the prevention and control of public health emergencies. Analyzing the effects of the integration of medical and preventive services on the prevention and control of public health emergencies enhances governance capacity for such emergencies and improves the prevention and control capabilities of integration, thereby protecting the lives and health.

To improve the prevention and control capabilities of the integration of medical and preventive services on public health emergencies in China, this study constructed an evaluation index system, calculated the comprehensive scores for 31 provincial-level administrative areas in China from 2018 to 2023 using the coupling coordination degree, and analyzed the effects of the integration on prevention and control of public health emergencies during the same period using a panel model. These findings provide scientific evidence to enhance health emergency response capabilities and improve national public health governance.

## Methods

### Data sources

Data on the integration of medical and preventive services and the effect of the prevention and control of public health emergencies in China were obtained from the China Health Statistics Yearbook (2019–2024) and the China Statistical Yearbook (2019–2024). The study covered data of 31 provincial-level administrative areas in mainland China (excluding Hong Kong, Macau, and Taiwan) from 2018 to 2023.

### Indicators

#### Explained variables

The development level of healthcare services directly impacts the effectiveness of infectious disease prevention, control, and treatment. The rational allocation of medical resources directly influences the early diagnosis and treatment of infectious diseases, and public health services can reduce the risk of infectious disease transmission. The coordinated development of medical and public health services enhances the effectiveness of infectious disease prevention and control. Therefore, the incidence of infectious diseases can be considered an overall reflection of health service outcomes. However, due to population mobility, infectious disease patients may concentrate in areas with higher medical standards, leading to significant regional disparities in infectious disease mortality rates. These disparities are unsuitable as evaluation indicators for the effectiveness of prevention and control. Therefore, the incidence rate of Class A and B infectious diseases is selected as the evaluation indicator.

#### Explanatory variables

In the context of the integration of medical and preventive services, medical services primarily refer to the diagnosis, treatment, and rehabilitation work carried out by medical professionals in healthcare settings for specific populations. Public health services mainly refer to a series of services provided by governments or relevant institutions to protect public health, including disease prevention and health education. When responding to public health emergencies, both medical and public health resources are utilized. Medical resources are primarily allocated to treatment, while public health resources are pre-dominantly directed toward prevention and surveillance. Integrating these two approaches enhances the health system's overall operational efficiency. Therefore, an evaluation index system for the integration of medical and preventive services was constructed from the perspectives of medical and public health resources. Medical resources refer to health resources within hospitals, including general hospitals, traditional Chinese medicine hospitals, integrated traditional Chinese and Western medicine hospitals, ethnic medicine hospitals, various specialized hospitals, and nursing hospitals. Public health resources refer to health resources within specialized public health institutions, including disease prevention and control centers, specialized disease prevention and control institutions, maternal and child healthcare institutions, health education institutions, emergency medical centers (stations), blood collection and supply institutions, health supervision agencies, and family planning technical service institutions under the jurisdiction of health authorities. Health resources refer to the social resources used for healthcare activities, including institutions, beds, and healthcare professionals (13). The number of institutions indicates the breadth of medical or public health services coverage and shows whether government health investments and health plans are being implemented according to administrative levels and functional roles. The number of beds reflects the scale and service capacity of medical or public health services, serving as a core indicator for measuring service provision capacity. Health technicians are the backbone of frontline care and represent the most dynamic component of health resources. Their professional competence, workload, and quality of work directly impact patient safety, treatment outcomes, and the patient experience. Licensed (assistant) physicians and registered nurses reflect the level of healthcare workforce allocation and are core indicators explicitly defined in national health plans that directly determine the accessibility and quality of healthcare services. Together, these indicators form a core framework for evaluating resource allocation, service capacity, and operational efficiency. This enables a comprehensive, objective assessment of the scale, efficiency, and equity of medical or public health services. To ensure comparability, the number of institutions, beds, health technicians, licensed (assistant) physicians, and registered nurses in hospitals and specialized public health institutions were selected as evaluation indicators (Table 1).

**Table 1:** Evaluation indicator system for the integration of medical and preventive services in China.

Numbers	Classification	Indicator	Indicator definition	Indicator direction
1	Explained variables	Incidence rate of Class A and B infectious diseases	Incidence rate of Class A and B infectious diseases per 100,000 population	—
2	Explanatory variables/medical resources	Number of institutions	Number of hospitals	Positive
3		Number of beds	Number of hospital beds	Positive
4		Health technicians	Health technicians in hospitals	Positive
5		Licensed (assistant) physicians	Licensed (assistant) physicians in hospitals	Positive
6		Registered nurses	Registered nurses in hospitals	Positive
7	Explanatory variables/Public health resources	Number of institutions	Number of public health institutions	Positive
8		Number of beds	Number of public health institution beds	Positive
9		Health technicians	Health technicians in public health institutions	Positive
10		Licensed (assistant) physicians	Licensed (assistant) physician in public health institutions	Positive
11		Registered nurses	Registered nurses in public health institutions	Positive
12	Control variables	Education level	Ratio of illiterate population to population aged 15 and above	—
13		Per capita GDP	Ratio of GDP to total population	—
14		Healthcare level	Ratio of health examination to total population	—
15		Urbanization level	Ratio of urban population to total population	—

#### Control variables

A higher the level of education leads to more effective utilization of healthcare resources, while population density may increase, thereby influencing the incidence of infectious diseases (14). A higher the per capita GDP increases an individual's capacity for healthcare expenditure, while greater economic development leads to increased interpersonal contact, affecting the effectiveness of public health emergency prevention and control. Higher levels of healthcare emphasize personal health and increase the utilization of medical resources, thereby facilitating the prevention and control of infectious diseases. A higher level of urbanization promotes the improvement of public health infrastructure, but it also leads to denser populations, which impacts the incidence of infectious diseases (15). Therefore, education level, per capita GDP, healthcare level, and urbanization level were selected as evaluation indicators (Table 1).

According to the “Law of the People's Republic of China on the Prevention and Control of Infectious Diseases”, Category A infectious diseases include two types: plague and cholera. Category B infectious diseases include 27 types, including monkeypox, novel coronavirus infection, severe acute respiratory syndrome (SARS), AIDS, viral hepatitis, poliomyelitis, human infection with new influenza subtypes, measles, hemorrhagic fever with renal syndrome, rabies, epidemic Japanese encephalitis, dengue fever, anthrax, bacterial dysentery and amoebic dysentery, tuberculosis, typhoid and paratyphoid fever, epidemic cerebrospinal meningitis, pertussis, diphtheria, neonatal tetanus, scarlet fever, brucellosis, gonorrhea, syphilis, leptospirosis, schistosomiasis, and malaria (16).

#### Regional division

Mainland China (excluding Hong Kong, Macau, and Taiwan) is divided into three regions: the eastern, central, and western regions. The eastern region includes 11 areas: Beijing, Hebei, Liaoning, Tianjin, Shandong, Shanghai, Zhejiang, Jiangsu, Guangdong, Fujian, and Hainan. The western region includes 12 areas: Guangxi, Inner Mongolia, Chongqing, Yunnan, Guizhou, Sichuan, Qinghai, Shaanxi, Gansu, Ningxia, Tibet, and Xinjiang. The central region includes eight areas: Heilongjiang, Anhui, Henan, Jiangxi, Jilin, Shanxi, Hubei, and Hunan.

### Research methods

The coupling coordination degree was used to calculate the comprehensive scores for the integration of medical and preventive services in 31 provincial-level administrative areas from 2018 to 2023. A panel model was used to analyze the effect of these scores on the prevention and control of public health emergencies during the same period.

### Coupling coordination degree

The coupling coordination degree was used to calculate the comprehensive scores for the integration of medical and preventive services in 31 provincial-level administrative areas from 2018 to 2023.

First, the data were normalized, and the entropy weight method was used to calculate the respective scores, S_1_ and S_2_, for medical resources and public health resources in China's integration of medical and public health services. The formula is:


$$\begin{array}{l} {\text{r}}_{\text{ij}}=\frac{x_{ij}-x_{min\left(j\right)}}{x_{max\left(j\right)}-x_{min\left(j\right)}} \end{array}$$ (1)

$$\begin{array}{l} {\text{r}}_{\text{ij}}=\frac{x_{max\left(j\right)}-x_{ij}}{x_{max\left(j\right)}-x_{min\left(j\right)}} \end{array}$$ (2)

$$\begin{array}{l} {\text{S}}_{\text{i}}=\overset{n}{\underset{i\;=\;1}{\sum }}\left(W_{j}\times X_{ij}\right) \end{array}$$ (3)

where i is the i-th year, j is the j-th indicator, n is the number of evaluation indicators, W_j_ is the weight of the indicator, and X_ij_ is the standardized value of the original data.

Second, the coupling degree (*C*) is calculated based on the respective scores of medical and public health resources, and the comprehensive coordination index (*T*) and the coupling coordination degree (*D*) are then calculated based on *C*. The coupling coordination degree reflects the coordination of the overall development of the integration of medical and public health services, with values ranging from 0 to 1, where a higher value indicates better coupling coordination (17). To reflect the integration of medical and public health resources, the coupling coordination index is used to represent the comprehensive score of the integration of medical and preventive services. The formula is:


$$\begin{array}{l} C\;=\;2\times \frac{\sqrt{S_{1}\times S_{2}}}{S_{1}+S_{2}} \end{array}$$ (4)

$$\begin{array}{l} D=\sqrt{C\times T} \end{array}$$ (5)

$$\begin{array}{l} T=\alpha S_{1}+\beta S_{2} \end{array}$$ (6)

Where α and β are undetermined coefficients. This study considers medical services and public health services to be equally important, so α = β = 0.5.

### Panel model

Using panel data from 31 provincial-level administrative regions in China from 2018 to 2023, a multiple linear regression model was constructed with the incidence rate of Class A and B infectious diseases as the explained variable, the comprehensive score of the integration of medical and preventive services as the explanatory variable, and educational level, per capita GDP, healthcare level, and urbanization level as control variables. Panel regression models typically include random-effects models, fixed-effects models, and pooled models (18). Fixed-effects models control for individual heterogeneity, focusing on internal variations. This makes them suitable for scenarios where individual effects are correlated with explanatory variables, or where the research focuses on causal relationships within a specific sample. Therefore, a fixed-effects model was selected for the analysis. Data analysis was performed using SPSS 22.0, where *P* < 0.05 indicates a statistically significant difference. The model is:


$$\begin{array}{l} \ln\text{ i}{\text{r}}_{\text{it}}=\alpha +\beta_{1}h_{it}+\beta_{2}X_{it}+\varepsilon_{it} \end{array}$$ (7)

where ir_it_ is the incidence rate of Class A and B infectious diseases in area i in period *t*, h_it_ is the comprehensive score of the integration of medical and preventive services in area i in period t, X_it_ is the set of control variables including education level, per capita GDP, healthcare level, and urbanization level, and ε_it_ is the error term.

## Results

### Weights of evaluation indicators for the integration of medical and preventive services in China

The weights for the ten evaluation indicators, which include the number of institutions, beds, health technicians, licensed (assistant) physicians, and registered nurses, were calculated using the entropy weight method for medical and public health resources in China from 2018 to 2023 (Table 2).

**Table 2:** Weights of evaluation indicators for the integration of medical and preventive services in China.

Year	Medical resources					Public health resources				
	Number of institutions	Number of beds	Health technicians	Licensed (assistant) physicians	Registered nurses	Number of institutions	Number of beds	Health technicians	Licensed (assistant) physicians	Registered nurses
2018	22.05	19.65	19.22	20.14	18.94	20.54	24.07	16.63	18.34	20.43
2019	21.67	19.76	19.31	20.23	19.03	19.05	24.72	17.30	17.68	21.25
2020	22.19	19.85	19.18	20.01	18.78	18.57	24.25	17.78	17.87	21.53
2021	22.39	19.75	19.15	20.03	18.69	16.17	25.22	18.25	18.60	21.75
2022	22.27	19.83	19.16	20.01	18.73	15.54	25.47	18.43	18.77	21.80
2023	22.09	19.82	19.20	20.07	18.83	15.10	25.48	18.38	19.49	21.56

### Comprehensive scores for the integration of medical and preventive services in China

The comprehensive scores for the integration of medical and preventive services in China from 2018 to 2023 were 0.558, 0.567, 0.574, 0.578, 0.577, and 0.579, respectively. These scores showed an overall increase. Areas such as Guangdong and Shandong had comprehensive scores above 0.94, indicating a relatively high level of integration. Areas such as Tibet, Qinghai, and Ningxia had comprehensive scores below 0.23, indicating a relatively low level of integration (Table 3).

**Table 3:** Comprehensive scores for the integration of medical and preventive services in China.

Regions	2018	2019	2020	2021	2022	2023
Beijing	0.433	0.436	0.435	0.435	0.430	0.429
Tianjin	0.257	0.262	0.260	0.258	0.257	0.257
Hebei	0.740	0.754	0.770	0.787	0.791	0.807
Liaoning	0.569	0.539	0.546	0.570	0.566	0.556
Shanghai	0.365	0.361	0.368	0.375	0.375	0.379
Jiangsu	0.731	0.744	0.764	0.784	0.767	0.766
Zhejiang	0.670	0.702	0.713	0.724	0.729	0.737
Fujian	0.520	0.528	0.539	0.544	0.538	0.544
Shandong	0.940	0.955	0.959	0.960	0.947	0.952
Guangdong	0.949	0.966	0.970	0.972	0.975	0.972
Hainan	0.248	0.248	0.259	0.266	0.266	0.262
Eastern region	0.584	0.590	0.598	0.607	0.604	0.606
Shanxi	0.544	0.534	0.544	0.550	0.543	0.541
Jilin	0.448	0.451	0.452	0.449	0.442	0.440
Heilongjiang	0.568	0.566	0.560	0.552	0.544	0.540
Anhui	0.592	0.600	0.635	0.638	0.664	0.672
Jiangxi	0.597	0.622	0.628	0.637	0.638	0.663
Henan	0.913	0.908	0.921	0.930	0.938	0.937
Hubei	0.703	0.703	0.707	0.718	0.719	0.731
Hunan	0.775	0.796	0.778	0.769	0.766	0.768
Central region	0.643	0.648	0.653	0.655	0.657	0.662
Inner Mongolia	0.481	0.489	0.488	0.487	0.491	0.498
Guangxi	0.659	0.677	0.689	0.678	0.678	0.670
Chongqing	0.440	0.444	0.449	0.453	0.457	0.457
Sichuan	0.791	0.816	0.831	0.843	0.841	0.843
Guizhou	0.550	0.562	0.577	0.601	0.606	0.612
Yunnan	0.608	0.640	0.659	0.667	0.668	0.671
Tibet	0.116	0.120	0.119	0.123	0.123	0.120
Shaanxi	0.603	0.609	0.635	0.609	0.605	0.604
Gansu	0.509	0.532	0.517	0.515	0.506	0.507
Qinghai	0.197	0.202	0.205	0.209	0.207	0.209
Ningxia	0.217	0.217	0.221	0.221	0.221	0.219
Xinjiang	0.459	0.479	0.485	0.486	0.475	0.481
Western region	0.469	0.482	0.490	0.491	0.490	0.491
Average score	0.558	0.567	0.574	0.578	0.577	0.579

Comprehensive scores for the integration of medical and preventive services in the eastern and central regions exceeded the Chinese average scores, while scores in the western region fell below it. The central region achieved the highest comprehensive score, followed by the eastern region, and the western region ranked third. In the eastern region, areas such as Guangdong and Shandong achieved a comprehensive score above 0.94 for the integration of medical and preventive services, while areas such as Hainan and Tianjin scored below 0.27, indicating greater development of the integration of medical and preventive services in Guangdong and lesser development in Hainan. In the central region, areas such as Henan achieved a comprehensive score above 0.90, while areas such as Jilin scored below 0.46, indicating relatively well-developed integration in Henan and poorer integration in Jilin. In the western region, areas such as Sichuan achieved a comprehensive score above 0.79, while areas such as Tibet and Qinghai scored below 0.21, indicating relatively well-developed integration in Sichuan and poorer integration in Tibet (Table 3).

### Pearson correlation analysis of evaluation indicators

Pearson correlation was used to assess the relationships among the evaluation indicators. The correlation coefficients between the incidence rates of Class A and B infectious diseases and the comprehensive score for the integration of medical and preventive services, per capita GDP, and urbanization level were −0.244, −0.377, and −0.474, respectively, all of which were significant at the 0.01 level. These results indicate a significant negative correlation between the incidence rates of Class A and B infectious diseases and the comprehensive score for the integration of medical and preventive services, per capita GDP, and urbanization level. The correlation coefficients between the incidence rates of Class A and B infectious diseases and the healthcare and educational levels were 0.233 and 0.355, respectively, these were significant at the 0.01 levels. These results indicate a significant positive correlation between the incidence rates of Class A and B infectious diseases and healthcare and educational levels. The correlation coefficient of urbanization and education levels is greater than 0.6, indicating a problem of multicollinearity. However, the correlation coefficients of healthcare level and per capita GDP are both less than 0.6, indicating no multicollinearity. Therefore, healthcare level and per capita GDP were selected as control variables (Table 4).

**Table 4:** Pearson correlation analysis of evaluation indicators.

Indicator	Incidence rate of Class A and B infectious diseases	Comprehensive scores for the integration of medical and preventive services	Healthcare level	Per capita GDP	Urbanization level	Education level
Incidence rate of Class A and B infectious diseases	1					
Comprehensive scores for the integration of medical and preventive services	−0.244^**^	1				
Healthcare level	0.233^**^	−0.123	1			
Per capita GDP	−0.377^**^	−0.031	0.201^**^	1		
Urbanization level	−0.474^**^	−0.026	0.073	0.818^**^	1	
Education level	0.355^**^	−0.393^**^	0.173^*^	−0.281^**^	−0.629^**^	1

^*^p < 0.05;

^**^p < 0.01.

### Regression results of the panel model for the integration of medical and preventive services in China

Model (1) served as the baseline model, reflecting the impact of comprehensive scores for the integration of medical and preventive services on the effectiveness of public health emergency response in China. Models (2) and (3) were derived from Model (1) by introducing control variables such as healthcare level, and per capita GDP.

Before the introduction of control variables, the comprehensive score for the integration of medical and preventive services was significant at the 0.01 level (*t* = −3.061, *p* < 0.01), with a regression coefficient of −612.315, indicating that the integration of medical and preventive services had a significant negative impact on the incidence rates of Class A and B infectious diseases. After introducing the control variables, the comprehensive score for integration of medical and preventive services was significant at the 0.05 level (*t* = −1.102, *p* < 0.05), with a regression coefficient of −227.428, indicating that the integration of medical and preventive services still had a significant negative impact on the incidence rates of Class A and B infectious diseases. Additionally, healthcare levels was positively correlated with the incidence rate of Class A and B infectious diseases, while per capita GDP was negatively correlated (Table 5).

**Table 5:** Regression results of the panel model for the integration of medical and preventive services in China.

Indicator	Model (1)	Model (2)	Model (3)
Comprehensive scores for the integration of medical and preventive services	−612.315^**^ (−3.061)	−635.693^**^ (−3.195)	−227.487^*^ (−1.102)
Healthcare level		0.279(1.822)	0.327^*^ (2.268)
Per capita GDP			−0.001^**^ (−4.645)
Intercept	558.387^**^ (4.907)	561.901^**^ (4.974)	428.228^**^ (3.897)
*R* ^2^	0.057	0.077	0.192
Sample size	186	186	186
Test	*F*_(1, 154)_ = 9.367, *p* = 0.003	*F*_(2, 153)_ = 6.414, *p* = 0.002	*F*_(3, 152)_ = 12.041, *p* = 0.000

^*^p < 0.05;

^**^p < 0.01.

### Regression results of the panel model for the integration of medical and preventive services in three regions of China

Models (4), (6), and (8) are baseline models that reflect the impact of the comprehensive score for the integration of medical and preventive services on the effectiveness of public health emergency prevention and control in the eastern, central, and western regions, respectively. Models (5), (7), and (9) are the models obtained after introducing control variables, such as healthcare level and per capita GDP. The comprehensive score for the integration of medical and preventive services in the eastern region was significant at the 0.01 level (*t* = −3.339, *p* < 0.01), with a regression coefficient of −703.139, indicating that such integration had a significant negative impact on the incidence rate of Class A and B infectious diseases. However, the central and western regions did not pass the significance level test, indicating that integration in these regions did not affect the incidence rate of Class A and B infectious diseases (Table 6).

**Table 6:** Regression results of the panel model for the integration of medical and preventive services in three regions of China.

Indicator	Model (4)	Model (5)	Model (6)	Model (7)	Model (8)	Model (9)
Comprehensive scores for the integration of medical and preventive services	−923.661^**^ (−4.651)	−703.139^**^ (−3.339)	−249.534(−1.046)	221.762(0.990)	−663.809(−1.387)	−47.909(−0.095)
Healthcare level		0.298(0.474)		1.226(1.529)		0.302(1.457)
Per capita GDP		0.002671		−0.002^**^ (−4.399)		−0.002^**^ (−2.800)
Intercept	729.759^**^ (6.143)	659.358^**^ (5.642)	357.317^*^ (2.294)	137.591(0.998)	573.168^*^ (2.466)	390.912(1.723)
*R* ^2^	0.286	0.383	0.027	0.370	0.032	0.179
Sample size	66	66	48	48	72	72
Test	*F*_(1, 54)_ = 21.635, *p* = 0.000	*F*_(3, 52)_ = 10.754, *p* = 0.000	*F*_(1, 39)_ = 1.095, *p* = 0.302	*F*_(3, 37)_ = 7.232, *p* = 0.001	*F*_(1, 59)_ = 1.924, *p* = 0.171	*F*_(3, 57)_ = 4.138, *p* = 0.010

^*^p < 0.05;

^**^p < 0.01.

## Discussion

Areas such as Guangdong and Shandong have comprehensive scores for integration of medical and preventive services above 0.94, while areas such as Tibet, Qinghai, and Ningxia have scores below 0.23, indicating significant regional disparities in the integration of medical and preventive services in China. We believe there are two reasons for this. On the one hand, the policy support varies by region, resulting in differences in the integration of medical and preventive services. For example, Guangdong has incorporated the integration of medical and preventive services into its health-first development strategy. The province has established tightly-knit medical consortiums, strengthened primary healthcare service networks, and promoted the sharing of medical and public health resources (19). Shandong has launched a national pilot program that integrates medical and preventive measures for infectious disease control. This program established a disease prevention and control system anchored by disease control institutions, supported by medical institutions, and grounded at the grassroots level (20). Areas such as Tibet, Qinghai, and Ningxia have inadequate fiscal capacity and long-standing functional divisions between medical institutions and public health institutions. These areas also have weak primary healthcare service capabilities, which constrain the development of the integration of medical and preventive services. On the other hand, the practice of integrated medical and preventive services varies across regions, leading to disparities in development levels. Areas such as Guangdong and Shandong provinces have actively implemented practical services, including family doctor contract services, tailored screening programs like pulmonary function tests, and the innovative “two prescriptions and one reminder” health management program (standard prescription + health prescription + health reminder), thereby promoting the integration of medical and preventive services (21). Areas such as Tibet and Qinghai have made slow progress in establishing county-level medical consortiums, sharing resources insufficiently, and providing inadequate telemedicine coverage. These factors have constrained the advancement of integration of medical and preventive services. Additionally, Henan (greater than 0.90) in the central region and Sichuan (greater than 0.79) in the western region had higher comprehensive scores for the integration of medical and preventive services, which indicates that these two areas are at an advanced level of integration development. Both Henan and Sichuan have clearly defined the public health functions of disease control institutions and medical institutions, promoting coordinated efforts between the two (22). They have also strengthened the informatics of medical institutions and the construction of tightly-knit county-level medical consortiums, promoting hospital information interconnectivity and the mutual recognition of examination results. This has driven the development of the integration of medical and preventive services. Among the eastern, central, and western regions, the central region had the highest comprehensive score for the integration of medical and public health services. This is due to the central region's relatively consistent level of integration, with most areas scoring above 0.50. The eastern region ranked in the middle in terms of the comprehensive score for the integration of medical and public health services. While regions such as Guangdong and Shandong have made significant progress, regions like Hainan and Tianjin have lagged behind, with scores below 0.27. These regions have long had a weaker public health system than a medical system. Their public health physician workforce is underdeveloped, and there is insufficient integration of information systems between the medical and public health sectors. Mechanisms for coordinated integration between medical and public health services have also developed slowly. The western region ranked third in the comprehensive score for the integration of medical and public health services. However, due to insufficient fiscal investment, a shortage of specialized technical personnel, and a lack of an effective coordination mechanism between medical and public health services, the integration of medical and public health services has not been fully implemented in the western region. Therefore, areas with lower development levels should improve the top-level design of their policies integrating medical and preventive services (23), promote resource sharing between medical and public health services, enhance the informatics of medical institutions, and implement practical services, such as family doctor contracts and chronic disease prevention and control services (24). These efforts would continuously advance the integration of medical and preventive services.

The comprehensive score for the integration of medical and preventive services in China was significant at the 0.05 level, indicating that the degree of integration has a significant effect on the effectiveness of the public health emergency response. We believe there are three reasons for this. First, the integration of medical and preventive services has strengthened the monitoring and early warning capabilities for public health emergencies. This integration breaks down information barriers between medical institutions and disease control institutions, enabling the real-time sharing and analysis of infectious disease data and laboratory data. This facilitates the early detection and reporting of legally notifiable infectious diseases (25). Medical institutions fulfill their public health responsibilities by enhancing public health surveillance and promptly identifying sources of infection and interrupting transmission routes. They play a crucial role in improving the effectiveness of public health emergency prevention and control. Second, the integration of medical and preventive services has improved the emergency response capabilities for public health emergencies. Medical institutions can swiftly meet emergency treatment demands with their existing personnel and equipment, enabling them to play a vital role in emergency response (26). During the COVID-19 pandemic, the full engagement of hospital medical resources shifted the frontline of treatment forward, becoming a key measure that yielded significant results in combating the outbreak (27). The establishment of public health prevention and control systems provides safeguards for promptly isolating suspected patients and blocking transmission sources (28). Through coordinated drills and the integration of resources from medical and public health institutions, response procedures for public health emergencies have improved, as have rapid response capabilities (29). Third, this integration has optimized health management for the public and enhanced their capacity to respond to public health emergencies. This integration has promoted health literacy and heightened public awareness of infectious disease control through practices such as family doctor contract services, chronic disease management, and personalized health guidance (30). Leveraging mechanisms such as resource consolidation, skills training, and information sharing, this integration has fostered the development of multidisciplinary professionals. Therefore, all areas should continue to advance the integration of medical and preventive services. They should also improve mechanisms for the coordinated development of medical institutions and public health institutions, promote the fulfillment of public health responsibilities by medical institutions, innovate practical approaches to integration, strengthen public health education and awareness campaigns (31), and enhance the integration's effectiveness in preventing and controlling public health emergencies.

The comprehensive score for the integration of medical and preventive services in the eastern region was significant at the 0.01 level, while the central and western regions did not reach significance, indicating regional differences in the effectiveness of the integration of medical and preventive services for controlling public health emergencies. We believe there are two reasons for this. First, the eastern region has achieved a relatively high level of integration of medical and preventive services, significantly enhancing the effectiveness of its public health emergency response. The eastern region has a large population and is relatively advanced in terms of socioeconomic development (32). It has an adequate allocation of medical and public health resources, as well as a well-established health services network. These factors enable the eastern region to provide high-quality health services and play a positive role in preventing and controlling public health emergencies (33). The eastern region began developing basic public health services earlier, shifting its disease burden from infectious diseases to chronic and age-related conditions, with the integration of medical and preventive services placing greater emphasis on health management and long-term care. The eastern region has actively innovated practical activities integrating medical and prevention. For example, it has established tightly-knit county-level medical consortiums, implemented intelligent disease surveillance, and incorporated health education throughout the entire disease treatment process. These efforts have effectively reduced the incidence of infectious diseases. Second, the central and western regions did not pass the significance level test, indicating that the integration of medical and preventive services has not significantly impacted public health emergencies. These regions have relatively weak healthcare foundations, slow development, and low health service standards. Health resources are concentrated in urban areas, while rural areas are relatively inadequate (34). There are significant disparities in health services across regions, which make achieving the synergistic integration of medical and preventive services difficult (35). The integration of medical and public health services in these regions aims to address weaknesses, strengthen grassroots capabilities, and improve accessibility. The primary goal is to ensure essential services are available and delivered effectively. The practice of integrating medical and prevention in central and western regions has developed slowly, with only a few areas, such as Henan and Sichuan, developing more effectively. Most regions, however, lag behind. Consequently, this integration has not significantly impacted the public health emergency response (36). Therefore, the eastern region should continue to innovate and develop integrated medical and preventive approaches. It should also improve health-centered management throughout the entire process and lifecycle of health services, and promote the coordinated development of medical and public health services (37). The central and western regions should actively learn from the eastern regions' innovative practices in integrating medical and preventive services. They should promote the tailored development of tightly-knit county-level medical consortiums, family doctor contract services, and chronic disease prevention and control. They should also swiftly build a collaborative response system for preventing and controlling public health emergencies through integrated medical and preventive efforts.

## Limitations

Although we used coupling coordination degree to calculate the comprehensive score for the integration of medical and preventive services in China from 2018 to 2023, and employed a panel model to analyze its effect on the prevention and control of public health emergencies during the same period, this study still has some limitations. First, although we established a one-to-one evaluation indicator system for medical and public health resources, the study results were inevitably affected by the indicator system. Second, the integration of medical and preventive services is a new development model influenced by regional policy differences, but this study did not analyze policy factors. Third, there are many forms of practice in the integration of medical and preventive services, but this study only described the main forms without summarizing or analyzing them. Fourth, we did not conduct interaction tests or subgroup comparisons to determine whether regional differences were statistically significant, which weakens the validity of the conclusions.

## Conclusions

To improve the prevention and control capabilities in the integration of medical and public health services for public health emergencies in China, we used the coupling coordination degree to calculate the comprehensive scores for this integration in 31 provincial-level administrative areas of China from 2018 to 2023, and used panel models to analyze the effects of this integration on the prevention and control of public health emergencies in China during the same period. The study found that the comprehensive scores for the integration of medical and preventive services in China from 2018 to 2023 were 0.558, 0.567, 0.574, 0.578, 0.577, and 0.579, respectively. Areas such as Guangdong and Shandong had comprehensive scores above 0.94, while areas including Tibet, Qinghai, and Ningxia had comprehensive scores below 0.23. Regional disparities in the integration of medical and preventive services in China were obvious. The comprehensive score for the integration of medical and preventive services in China showed statistical significance at the 0.05 level (*t* = −1.102, *p* < 0.05), with a regression coefficient of −227.428. This indicates that the integration of medical and preventive services has a significant negative impact on the incidence rates of Class A and B infectious diseases. The comprehensive score in the eastern region was statistically significant at the 0.01 level (*t* = −3.339, *p* < 0.01), with a regression coefficient of −703.139. The central and western regions did not pass the significance level test. The effect of the integration of medical and preventive services on the prevention and control of public health emergencies varies by region. We suggest that regions should refine the top-level design of integrated medical and preventive policies, promote resource sharing between medical and public health services, and implement practical services such as tightly-knit medical consortiums, family doctor contract services, and chronic disease prevention and control. These measures will further enhance the effects of integrated medical and preventive services on the prevention and control of public health emergencies.

## Data Availability

The raw data supporting the conclusions of this article will be made available by the authors, without undue reservation.

## References

[1] Pengfei Z Jinghua G. Evaluation of China's public health system response to COVID-19. J Global Health. (2021) 11:05004. doi: 10.7189/jogh.11.05004 33643637 PMC7897426

[2] General Office of the State Council of China. Guiding Opinions on promoting High-Quality Development of Disease Prevention and Control Issued by the General Office of the State Council of China (in Chinese). (2023) Available online at: https://www.gov.cn/zhengce/zhengceku/202312/content_6922484.htm (Accessed March 10, 2016).

[3] Faguang W Xue F Ang S Ribesh K Jin H. How is the medical service efficiency in China? An empirical analysis using stochastic frontier approach and gravity models. Int J Health Plan Manag. (2022) 37:2949–63. doi: 10.1002/hpm.3534 35775602

[4] Jie Z Jianzhong Z. Effective policy research of county and township health sector integration in China: empirical evidence from the difference-in-differences model. Soc Sci Med. (2024) 348:116797. doi: 10.1016/j.socscimed.2024.116797 38547805

[5] Mingyuan Z Lishu Y Baoshan Q Yun Y Gongbo W Chen L. Physical-medical integration policies and health equity promotion in China: a text analysis based on policy instruments. Int J Equ Health. (2024) 23:266. doi: 10.1186/s12939-024-02327-9 39696574 PMC11656552

[6] Madison KR Rebecca EC Kirsten C Jonathan P Gail K Pamela S . Public health surveillance of behavioral health emergencies through emergency medical services data. Prehospit Emer Care. (2022) 26:792–800. doi: 10.1080/10903127.2021.1973626 34469269

[7] Anne CJ Genevive RM Donna L Sabine J Rachel B John DC . Pandemic response officers: integration between medical, public health, and higher education systems to expedite prevention and response. J Pub Health Manag Prac. (2023) 29:556–62. doi: 10.1097/PHH.0000000000001704 36727770

[8] Gupteswar P Caragh B Ann T Surekha G. The dynamics of TCAM integration in the Indian public health system: medical dominance, countervailing power and co-optation. Soc Sci Med. (2021) 286:114152. doi: 10.1016/j.socscimed.2021.114152 34465489

[9] Liana P Julie S Lily L Jorge D Lillie F Geoff W . Community health work and social work collaboration: integration in health care and public health settings: a conceptual framework. J Ambul Care Manag. (2024) 47:187–202. doi: 10.1097/JAC.0000000000000498 38775666

[10] Zhen W Huiyi T Dongjian X Jiaying C Yaqi H Xiaohe W . Influencing factors and symbiotic mechanism of the integration of medical care and disease prevention during the covid-19 pandemic: a cross-sectional survey of public hospital employees. International J Environ Res Public Health. (2022) 20:241. doi: 10.3390/ijerph20010241 36612563 PMC9819979

[11] Kunhe L Xiao L Li X Fei L. Sanming model of medical service integration: impact on medical expenditures, service provision, and resource allocation in China. Risk Manag Healthcare Policy. (2025) 18:205–16. doi: 10.2147/RMHP.S503613 39834651 PMC11745052

[12] Sheng-Hu T Yu C Tao-Zhen B. Key influencing factors of vertical integration of electronic health records in medical consortiums. Int J Med Informat. 2023,170:104959. doi: 10.1016/j.ijmedinf.2022.104959 36542900

[13] Feng Q Ao YB Chen SZ Martek I. Evaluation of the allocation efficiency of medical and health resources in China's rural three-tier healthcare system. Public Health. (2023) 218:39–44. doi: 10.1016/j.puhe.2023.02.009 36965462

[14] Yu X Xiaohui R. Determinants of province-based health service utilization according to Andersen' s behavioral model: a population-based spatial panel modeling study. BMC Public Health. (2023) 23:985. doi: 10.1186/s12889-023-15885-4 37237347 PMC10224305

[15] Hao W Guoliang M Hui L. Determinants of equitable public health human resource allocation in China: a multidimensional analysis using RIF-I-OLS decomposition. Human Res for Health. (2025) 23:54. doi: 10.1186/s12960-025-01019-x 41094470 PMC12522753

[16] The National People's Congress of the People's Republic of China. Law of the People's Republic of China on the Prevention and Control of Infectious Diseases. (in Chinese). Available online at: http://www.npc.gov. cn/npc/c2/c30834/202504/t20250430_445085.html?sign=ABZ0cnNfd2NtX3ByZXZ pZXdfYWNjZXNzAAAH6QAAAAQAAAAIAAAACQAAABcAAAAn (Accessed April 04, 2016).

[17] Mengyuan M Leiyu S Wanzhen X Qiuli Z Junqing L Shengwu L . Coupling coordination degree of healthcare resource supply, demand and elderly population change in China. Int J Equity in Health. (2024) 23:147. doi: 10.1186/s12939-024-02236-x 39049064 PMC11270932

[18] Guangqin L Lingyu L Dan L Jiahong Q Hongjun Z. Effect of PM2.5 pollution on perinatal mortality in China. Sci Reports. (2021) 11:7596. doi: 10.1038/s41598-021-87218-7 33828199 PMC8026972

[19] He Y-T Zhang Y-C Wu R-K Wen H Wang R-N He L-X . Dynamic evolution and spatial difference of public health service supply in economically developed provinces of China: typical evidence from Guangdong Province. BMC Health Serv Res. (2024) 24:23. doi: 10.1186/s12913-023-10444-4 38178099 PMC10768127

[20] Qianqian L Shusheng H Xiaoyuan Q Aitian Y. The status of health promotion lifestyle and its related factors in Shandong Province, China. BMC Public Health. (2021) 21:1146. doi: 10.1186/s12889-021-11152-6 34130669 PMC8207564

[21] YingJie G YaQiong W XiaoLin Y GuanNan S Cong C Xiang G. Development prospects of “Internet + medical and health services” in Shandong: application status and public acceptance. Front Public Health. (2025) 13:1589224. doi: 10.3389/fpubh.2025.1589224 40843430 PMC12364942

[22] Rongmei L Qiuping Z Wenyong D Dan G Zhanlei S Yi L . Assessing public health service capability of primary healthcare personnel: a large-scale survey in Henan Province, China. BMC Health Serv Res. (2024) 24:627. doi: 10.1186/s12913-024-11070-4 38745226 PMC11094852

[23] Yibeltal A Charles FG Simon R Remco P Dereje GG Wim Van D. Analysis of the COVID-19 pandemic: lessons towards a more effective response to public health emergencies. Globalization and Health. (2022) 18:10. doi: 10.1186/s12992-022-00805-9 35120537 PMC8815718

[24] Yanni Y Yue Z Anling X. Information interaction and social support: exploring help-seeking in online communities during public health emergencies. BMC Public Health. (2023) 23:1250. doi: 10.1186/s12889-023-16151-3 37370074 PMC10304684

[25] Sileshi DS Pien VZ Getinet A Fantu MA Mark S. Information sharing across institutions: Practices and barriers during public health emergencies in Ethiopia. Int J Med Informat. (2024) 186:105439. doi: 10.1016/j.ijmedinf.2024.105439 38564958

[26] Qian L Lin W Bangfang W Hongzhou L. The COVID-19-designated hospitals in China: preparing for public health emergencies. Emerg Microb Infect. (2021) 10:998–1001. doi: 10.1080/22221751.2021.1931467 33993856 PMC8183501

[27] Tao L. Trans-regional medical support in public health emergencies: a case study of wuhan in the early COVID-19 pandemic in China. Risk Manag Healthcare Policy. (2022) 15:677–83. doi: 10.2147/RMHP.S346556 35449543 PMC9017699

[28] PreciousWC Kelly KB Leah A Marina DR Daniel JD Tricia K . Equity in epidemic response: an action-oriented framework for guiding public health in equitable responses to major infectious disease emergencies. Int J Equity Health. (2025) 24:69. doi: 10.1186/s12939-025-02433-2 40075487 PMC11900141

[29] Sicheng H Zibo L Xinqi L Lin L Feng R Wenhua M . Establishment of a no-notice drill mode evaluation system for public health emergencies. PLoS ONE. (2022) 17:e0266093. doi: 10.1371/journal.pone.0266093 35377910 PMC8979443

[30] Yazhuo G Ying C Yin L Fangfang Z Xuehua Z. Urban residents' self-rescue in response to public health emergencies in China: a qualitative study. BMC Public Health. (2023) 23:2520. doi: 10.1186/s12889-023-17442-5 38104101 PMC10724934

[31] Laura JF Sara JV Mary L Robin ES Joie DA. Integrating behavioral health into monitoring and surveillance during public health emergencies: challenges and opportunities. Disaster Med Public Health Preparedness. (2024)18:e132. doi: 10.1017/dmp.2024.127 39291362 PMC11483156

[32] Zhun Y Tingling X Jing Y Shicheng Y Maigeng Z Han L . Comprehensive assessment of resources for prevention and control of chronic and non-communicable diseases in China: a cross-sectional study. BMJ Open. (2023) 13:e071407. doi: 10.1136/bmjopen-2022-071407 37474175 PMC10360424

[33] Liu M Shen W Xin Z. A study on the evolution of tripartite collaborative prevention and control under public health emergencies using COVID-19 as an example. Sci Rep. (2024) 14:3135. doi: 10.1038/s41598-024-53601-3 38326403 PMC10850330

[34] Shaoyang Z Yueqin W Yuxiao C Mei Z. Healthcare resource allocation and patient choice: evidence from rural China. Int J Equity in Health. (2025) 24:87. doi: 10.1186/s12939-025-02450-1 40165191 PMC11959753

[35] Jisha Z Jing L Guolei C Chunyan Z Lianlian L Taijia M. Aspects of public health development in China's western region. Geospatial Health. (2024) 19. 10.4081/gh.2024.1252 38619395

[36] Yueyang W Ruigang D Xinyu G. Exploring the integration of medical and preventive chronic disease health management in the context of big data. Front Public Health. (2025) 13:1547392. doi: 10.3389/fpubh.2025.1547392 40302775 PMC12037625

[37] Jean-PU Ingrid M Pierre DP Michel R. Integrating clinical and public health knowledge in support of joint medical practice. BMC Health Serv Res. (2020) 20:1073. doi: 10.1186/s12913-020-05886-z 33292211 PMC7724788

